# UVC photon-induced denaturing of DNA: A possible dissipative route to Archean enzyme-less replication

**DOI:** 10.1016/j.heliyon.2019.e01902

**Published:** 2019-06-18

**Authors:** Karo Michaelian, Norberto Santillán Padilla

**Affiliations:** a Department of Nuclear Physics and Application of Radiation, Institute of Physics, UNAM, Cto. Interior de la Investigación Científica, Ciudad Universitaria, Cuidad de México, C.P. 04510, Mexico; b Faculty of Science, UNAM, Cto. Interior de la Investigación Científica, Ciudad Universitaria, Cuidad de México, C.P. 04510, Mexico

**Keywords:** Molecular biology, Biochemistry, Origin of life, DNA, RNA, DNA denaturing, Irreversible thermodynamics, Homochirality, Dissipation, Enzyme-less replication

## Abstract

Non-equilibrium thermodynamics is a relevant framework from within which to address formidable difficulties encountered in explaining the origin of life; from molecular synthesis and complexation, enzyme-less proliferation, to evolution (including the acquisition of homochirality and information). From within this framework we have proposed that the origin of life was the origin of the dissipative structuring of organic pigments which became the fundamental molecules of life (e.g. RNA and DNA) proliferated through autocatalytic photochemical reactions under the thermodynamic imperative of dissipating the imposed UVC solar photon flux available at the Archean surface. Here we present experimental evidence demonstrating that the absorption and dissipation of UVC light by synthetic DNA of 25 base pairs (and also natural salmon sperm DNA) over a range of temperatures, including below their melting temperature, leads to denaturing. Since denaturing is a non-trivial step on route to enzyme-less replication, our data suggest the possibility of a dissipative route to DNA replication at the origin of life. Such a dissipation-replication relation provides a simple mechanism for the early accumulation of both homochirality and information. Possible mechanisms of UVC photon-induced denaturing of DNA are discussed.

## 1 Introduction

Non-equilibrium thermodynamics indicates that the origin, persistence, and evolution of any irreversible process require the dissipation of a generalized thermodynamic potential. We have conjectured [1, 2, 3, 4, 5, 6, 7, 8, 9, 10, 11, 12, 13] that the early synthesis, complexation, proliferation and evolution of the fundamental molecules of life (those common to all three domains; bacteria, eukarya, and archea) were driven by the dissipation of the long-wavelength (220–290 nm) part of the UVC solar photon spectrum prevailing for at least 1,000 million years at Earth's surface throughout the Archean [14, 15]. Photons from within this region of the solar spectrum have enough free energy to reconfigure covalent bonds of carbon based organic molecules, but not enough energy to disassociate these. From this perspective, molecular synthesis can be understood as microscopic dissipative structuring of pigments [6, 9, 13] and proliferation can be understood as autocatalytic photochemical replication of these [5], both driven by the dissipation of the Archean solar long-wavelength UVC photon flux; a process still relevant today for organic pigment synthesis and proliferation, albeit at visible wavelengths of lower photon energy, thus requiring routes of much greater biochemical complexity [6, 7, 8].

Indeed, many of the fundamental molecules of life including nucleotides, amino acids, enzymes, vitamins, cofactors, stacked protoporphyrins, and conjugated fatty acids, all absorb and dissipate photons strongly within the 220–290 nm long wavelength UVC region [10], precisely that wavelength region where Sagan [14] and later Cnossen et al. [15] established the probable existence of an Archean atmospheric window.

Evolution of greater molecular complexity, for example the polymerization of UVC-activated nucleotides into oligomers and the association of these with UVC antenna molecules such as the aromatic amino acids, as well as with other fundamental molecules of life, also has a thermodynamic explanation since fluctuations leading to molecular reconfigurations which increase the dissipation of the externally imposed generalized chemical potential are favored by the current-fluctuation theorem. That such a theorem is indeed operating in nature has been established through ample empirical evidence indicating an increase in the photon dissipation of the biosphere over time [8]. For example, polymerization of the nucleotides could have provided a scaffold for the stereochemical attachment of antenna-like UVC absorbing molecules, such as the aromatic amino acids, allowing resonant energy transfer between these excited molecules and specific coding DNA or RNA oligomers which could then dissipate the free-energy on a subpicosecond time scale through their conical intersection into vibrational energy of the surrounding water molecules [16]. Polymerization of the nucleotides would also have provided enhanced stability against hydrolysis [17] (particularly at the high surface temperatures of the early Archean ∼80–85 °C [18, 19]) and a template for reproduction, i.e. the copying of the most efficient photon dissipating DNA/RNA + antenna complexes.

UVC chromophores have conjugated carbon bonds leading to de-localized electrons which not only gives them their extraordinary light absorbing properties but also their planer aromatic structures and thus chemical affinity to RNA and DNA through stacking interactions by intercalating between consecutive base pairs. Many also have chemical affinity to the secondary or tertiary structures of RNA and DNA [20]. The UVC absorbing amino acids, including the aromatic tryptophan, tyrosine, phenylalanine, as well as the photon-induced charge transfer amino acids histidine, cysteine and lysine, have particularly strong chemical affinity to their DNA codons or anti-codons [21], suggesting not only a stereochemical era [21, 22, 23, 24], but also a UVC dissipative era for incipient life [2, 3, 4, 6, 9, 10].

A difficult problem related to the origin of life is explaining enzyme-less reproduction of RNA or DNA [25]. We have proposed UVC photon-induced breaking of native RNA or DNA intra-strand hydrogen bonds, promoted by light-induced dimerizations and charge transfers, and local heating resulting from the internal conversion of photons, once the ocean surface temperature descended below a value allowing hybridization of single strands [2, 3, 6, 9]. This enzyme-less denaturing would have permitted subsequent extension during overnight dark periods facilitated by Mg^2+^ ions [25] and UV-activated phosphorylated nucleotides [26]. This proposed ultraviolet and temperature assisted replication (UVTAR) is similar to polymerase chain reaction (PCR) [27] but in which temperature cycling is substituted by diurnal UVC light cycling, and in which Mg^2+^ ions, or their complexes with some hypothetical common primordial molecule, played the role of the contemporary extension enzyme “polymerase”.

Here we present experimental evidence for UVC photon-induced denaturing of short 25 base pairs (bp) synthetic DNA and long (∼100 kbp) salmon sperm DNA. A comparative analysis of the hyperchromism in our extinction data for double and single strand 25 bp DNA, as well as an analysis of the absorption difference spectra obtained over a UVC light on-sample period for different fixed temperatures, suggests that UVC photon-induced denaturing is of sufficient magnitude to be of relevance to a possible dissipative route to enzyme-less replication of nucleic acids at the origin of life.

## 2 Method

Complementary synthetic DNA oligonucleotides of 25 base pairs and salmon sperm DNA samples of varied lengths (∼100 ± 70 kbp) obtained through centrifuge shearing were provided by the Institute of Cellular Physiology at the National Autonomous University of Mexico (IFC-UNAM). The synthetic oligos were designed to be free of adjacent thymine to reduce the complications of photon-induced cyclobutane pyrimidine dimer (CPD) formation, known to induce local denaturing [28]. The oligos were also designed to have convenient denaturing and priming temperatures in order to facilitate a possible UVTAR mechanism operating together with a probable small diurnal Archean ocean surface temperature cycling (∼3 °C) as occurs today at the ocean surface [29]. The sequence chosen for the 25bp synthetic DNA was (5′-3′), CTATGGAGCGGATATACCATGGACG, containing 52% GC content.

To form the double helices, complementary oligos at equal concentration were mixed in a Dulbecco PBS buffer (pH 7.3) solution containing 2.7 mM potassium chloride (KCl), 136.9 mM sodium chloride (NaCl), 1.5 mM potassium phosphate monobasic (KH_2_PO_4_) and 8.9 mM sodium phosphate dibasic (Na_2_HPO_4_). The salmon sperm DNA was dissolved in purified water (Mili-q).

The resulting concentrations of double helix DNA were determined from their absorption at 260 nm to be 1.56 and 0.00023 μM for the 25 bp and salmon sperm DNA respectively (assuming average lengths of 100 kbp for the latter).

3.5 ml and 2.0 ml of the corresponding solution of synthetic 25 bp and salmon sperm DNA respectively was placed into a tightly stoppered 3.5 ml quartz cuvette of 1 cm light path length. The cuvette was placed inside a precise (±0.01 °C) Ocean Optics^@^ temperature control unit operating via the Peltier effect with water flow stabilization of the temperature for precise and rapid temperature control when removal of heat from the solution was required. The temperature was monitored via a probe located in one of the four supporting towers of the cuvette and in direct contact with it. A magnetic stirrer provided assurance of temperature equilibration throughout the volume, prevented DNA sedimentation, and assured homogeneous passage of all DNA through the approximately 3.7 mm diameter light beam on-sample.

UV light from either a 3.8 W (9.4 μW on-sample) or a 26 W (217 μW on-sample) deuterium source, covering continuously the range 215–850 nm (see Supplementary Content, Fig. S1) was defocused onto the DNA sample via UV light resistant 30 cm long optical fiber of 600 μm diameter and a quartz lens. After passing through the sample, the surviving light was collected by a second lens and focused onto a similar optical fiber which fed into an Ocean Optics^@^ HR4000CG charge-coupled device spectrometer covering the range 195–1120 nm with a resolution of 0.3 nm. Spectrometer integration times were of the order of 1 s for the low intensity lamp and 20 ms for the high intensity lamp, and the raw data were software averaged over five wavelength bins (1.5 nm).

**Fig. 1:**
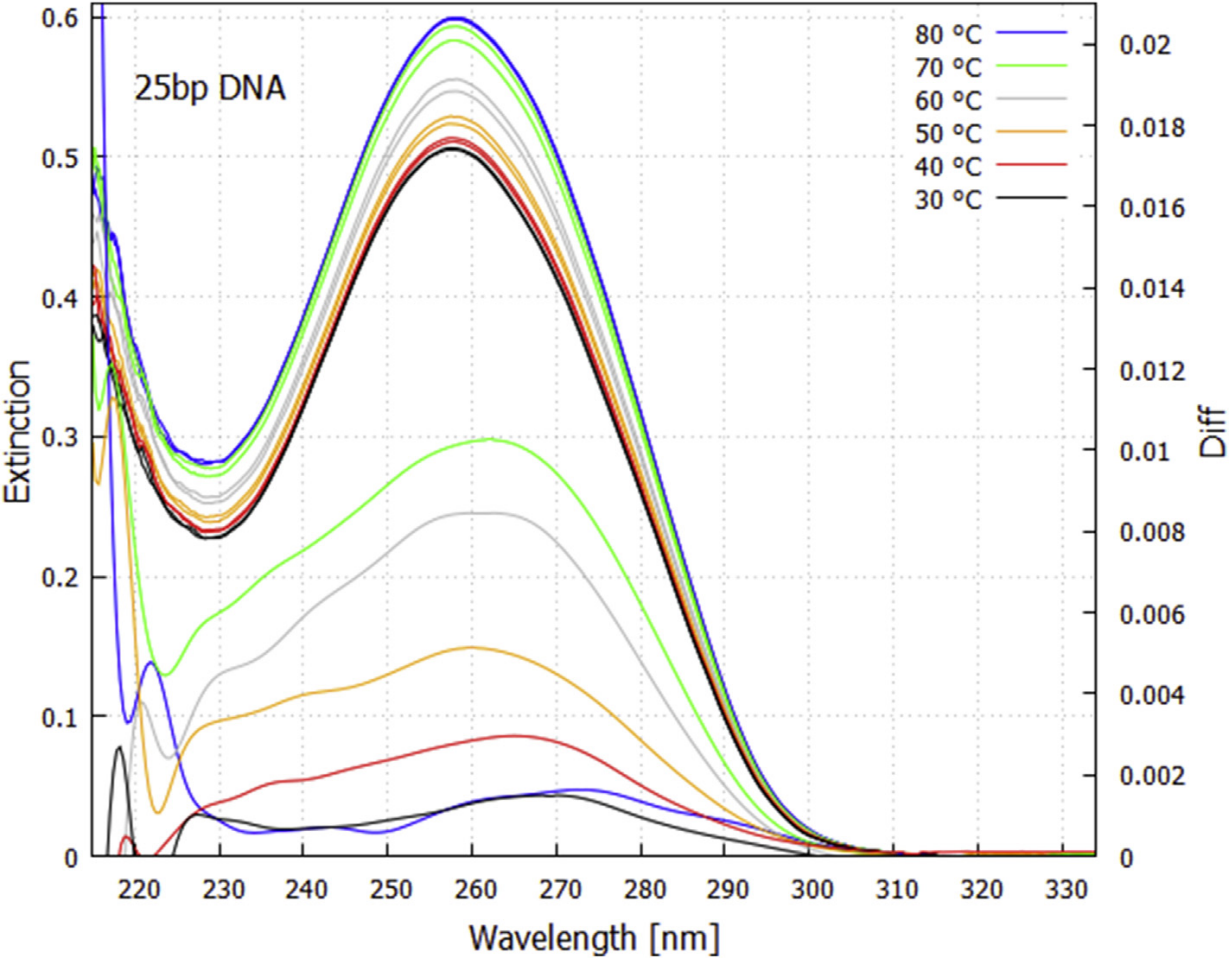
Extinction as a function of wavelength and temperature for the 25 bp DNA in PBS buffer and the corresponding thermal *difference spectra* (lower curves, right y-axis) obtained by subtracting the lower temperature extinction curve from the higher temperature curve for a 3 °C bin centered at the specified temperature. The difference spectra have been smoothed with a 2000 point Bezier function.

Extinction (absorption plus scattering) spectra, E(λ), were obtained by comparing the measured intensity spectrum, I(λ), of the light through the DNA sample with a reference spectrum, $I_{R}\left(\lambda \right)$, obtained at 55 °C with a similar quartz cuvette containing only either PBS buffer or purified water as required, but no DNA. A dark spectrum, $I_{D}\left(\lambda \right)$, obtained with a shutter blocking the deuterium light on sample, was subtracted from the spectra to correct for stray light and electronic noise. Spectrometer baseline drift over time BD(t) was accounted for by normalizing counts in the spectrometer for wavelengths 208.0–208.2 nm (where no light from the deuterium lamps was detected, see Supplementary Content, Fig. S1) to that of the dark spectrum. Lamp intensity and spectrometer gain drift over time LD(t) was accounted for by normalizing spectra to counts in the wavelength region of 314.8–315.9 nm (the shortest wavelength corresponding to negligible DNA absorption, see Fig. 1) to that of the reference spectrum. Extinction spectra were thus calculated as;

(1) $$E\left(\lambda ,t\right)=-log_{10\;}\left(\frac{\left(I\left(\lambda \right)*BD\left(t\right)-I_{D}\left(\lambda \right)\right)*LD\left(t\right)}{\left(I_{R}\left(\lambda \right)*BD\left(t\right)-I_{D}\left(\lambda \right)\right)}\right).$$

The synthetic 25 bp DNA sample was heated rapidly (5 °C/min) from room temperature to 85 °C and held there for approximately 10 minutes to ensure complete denaturing. The temperature of the sample was then lowered slowly (0.3 °C/min) to that desired for the particular run with the shutter blocking the light. The long salmon sperm DNA sample was brought directly to the temperature of the run from ambient temperature (∼24 °C). Runs consisted of allowing the sample to equilibrate (between denatured single and natured double strands) for approximately 30 minutes after arriving at the given temperature of the run, prior to retracting and inserting the shutter to cycle the deuterium light on and off through the sample for regular time periods of 60 minutes (or 30 minutes for the natural DNA) while continuously monitoring the wavelength dependent extinction, Eq. (1).

Data Availability: The datasets generated during and/or analyzed during the current study are available from the corresponding author on reasonable request.

## 3 Results

Fig. 1 shows the extinction spectra obtained with the intense deuterium light source for the 25 bp synthetic DNA delimited by a 3 °C bin centered at the specified temperatures. The extinction peak at 258 nm is due predominantly to absorption on the nucleic acid bases but also includes a small amount (<1%) of Rayleigh and Mie scattering. An increase in extinction with temperature is observed, known as hyperchromism, which results from denaturing allowing the initially tightly stacked nucleobases to become more exposed to the UVC light [30].

The lower curves of Fig. 1 are the corresponding thermal *difference spectra* obtained by subtracting the lower temperature extinction curve from the higher temperature curve for the 3 °C bin centered at the specified temperature.

**Fig. 2:**
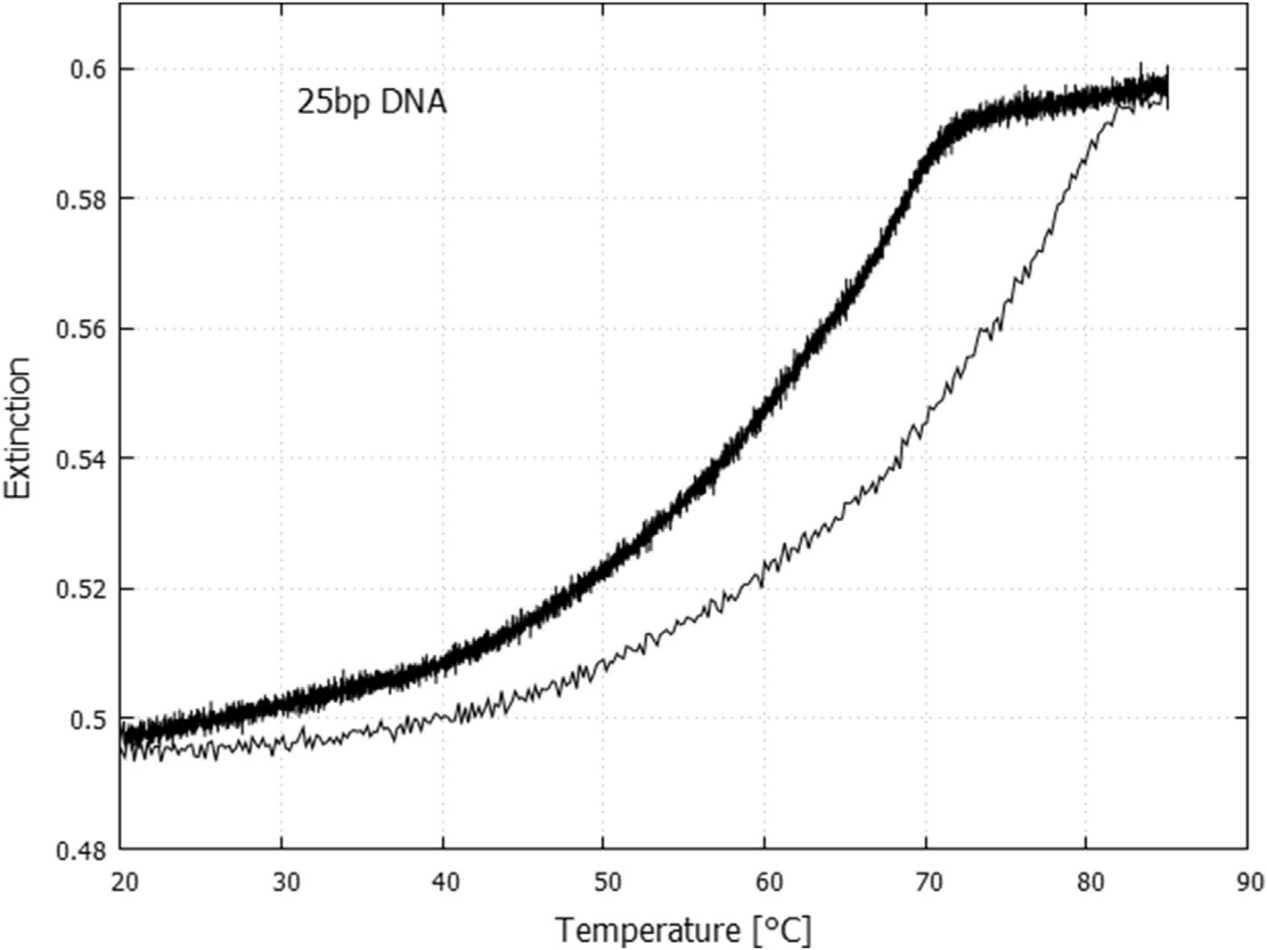
The denaturing curve for the 25 bp synthetic DNA in PBS buffer obtained by averaging the extinction over wavelengths 255–262 nm (centered on the peak at 258 nm, Fig. 1). The melting temperature, as determined by the half-way point of the hyperchromic rise is 59.4 °C, and by the inflexion in the slope is 69.1 °C. The well known hysteresis is observed between the curve for a rapid increase in temperature (5 °C/min – thin line) to 85 °C and that for a slow decrease in temperature (0.3 °C/min – thick line) to 20 °C.

Fig. 2 shows the temperature denaturing curve obtained for the synthetic 25 bp DNA.

**Fig. 3:**
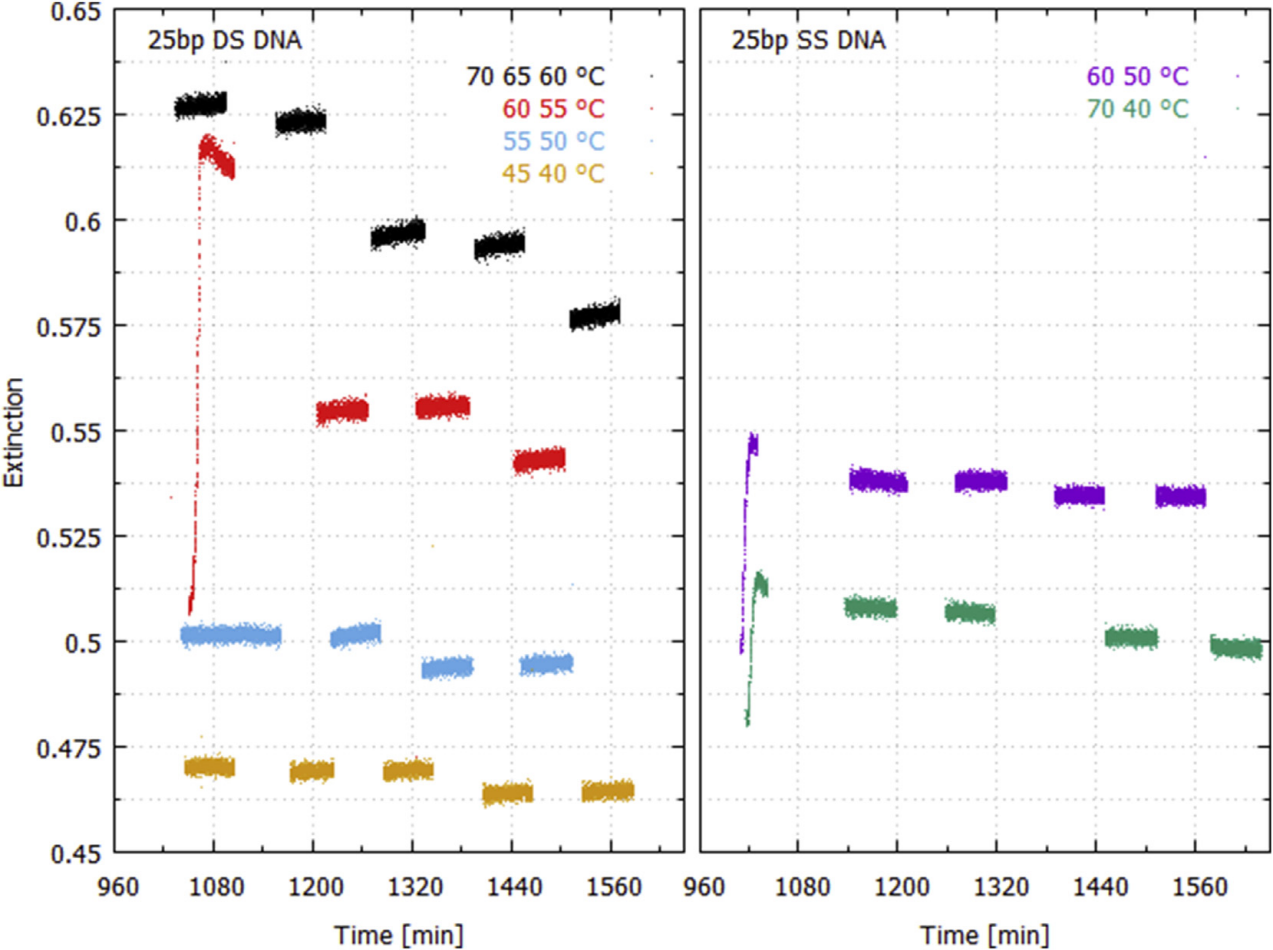
Extinction averaged over wavelengths 250–266 nm as a function of time (minutes since midnight) for double strand (left) and single strand (right) 25 bp DNA while cycling the deuterium light on and off through the sample for periods of one hour. There are at least 2 repetitions at each temperature. Traces of a given color represent a run of a particular day normalized to the particular dark spectrum for that run and compared to a reference spectrum common to all runs. An increase in extinction is observed during the light-on periods for double strand DNA, but not for single strand.

Fig. 3 plots the extinction, Eq. (1), averaged over wavelengths 250–266 nm, for the duration of the light cycling runs for the synthetic 25 bp double strand (left panel) and single strand (reverse complement 5′to 3′, right panel) DNA sample at different fixed temperatures. For the double strand DNA, the extinction always increases during the one hour light-on periods (particularly noticeable at the higher temperatures) and decreases or stays constant during the one hour light-off periods. Since, as a result of the hyperchromism, absorption increases on denaturing, this is tentative evidence for UVC light-induced denaturing of the double strand DNA. The decrease in extinction during the light-off periods is tentative evidence for dark period renaturing. This does not occur for the single strand DNA samples (right panel), which, in fact, show a small decrease in extinction during the light-on periods.

Before considering these results as evidence for UVC light-induced denaturing, other physical or instrumental effects which may have influenced the result should be considered. A change in extinction may be due to any combination of the following;

1) absorption hyperchromicity due to light-induced denaturing (the sought after effect),
2) Rayleigh or Mie scattering due to light-induced denaturing changing the number and shape of the scattering centers,
3) UV-induced damage to DNA, mostly in the form of cyclobutane pyrimidine dimers (CPDs) and their (6-4) products which reduce absorption,
4) evaporation of the solvent, thereby increasing the concentration of the DNA solute,
5) sedimentation of the DNA, thereby decreasing the concentration of the DNA solute exposed to the light beam,
6) variation over time of the light output from the light source, or variation of the spectrometer response.

Points 1) and 2) are the sought after effect for which the experiment was designed. Regarding point 3), for our wavelengths of interest, the most common UV-induced damage is the formation of CPD dimers (cross section peaking at 270 nm) which can be photo-reverted by shorter wavelength light (cross section peaking at 239 nm). Such “damage” is therefore not permanent and, in fact, contributes to reversible UVC light-induced denaturing (see Discussion and Supplementary Content).

Concerning point 4), even though the cuvette was tightly stoppered, a small amount of evaporation of the solvent could have occurred, particularly during the high temperature runs, leading to an increase in concentration and hence absorption. However, the evaporation rate should be independent of the light-on or -off condition and also independent of whether double or single strand DNA was in the sample holder. Fig. 3 shows that the extinction dropped (remained constant) over the light-off periods for the double (single) strand DNA and did not increase during the light-on periods for the single strand DNA. This suggests that evaporation had a negligible influence on our data. Concerning point 5), sedimentation of the DNA would lead to a reduction in concentration and therefore a reduction in extinction. However, sedimentation was avoided during the experiment by maintaining the stirrer at maximum setting for all data runs.

Concerning point 6), the light-drift convoluted with the detector response was compensated for in the off-line analysis by normalizing the light through sample data to that of the reference spectrum at the shortest wavelengths for which no DNA absorption was detected, corresponding to the interval of 314.8–315.9 nm (see Fig. 1), giving the correction factor LD(t) in Eq. (1). The light drift convoluted with the detector response, after allowing ½ hour for lamp warm-up, is given in Supplementary Content, Fig. S2. The drift is uniform over the 5 hour run with little dependence on wavelength and is not affected by changing the shutter condition. The convoluted drift is influenced mostly by variations of the line voltage and ambient temperature, as determined by the manufacturer of both these instruments [31].

### 3.1 Difference spectra for UVC photon-induced denaturing

After controlling, or correcting, for the instrumental effects mentioned above, the increase in extinction during the light-on periods observed in Fig. 3 for double strand DNA but not for single strand DNA is therefore strong indication of UVC light-induced denaturing. However, more conclusive evidence for denaturing can be obtained by studying the wavelength dependence of this increase in extinction during the light-on periods. To this end we determined the UVC light-induced *difference spectrum* [32], obtained by subtracting the extinction spectrum obtained at the start of a prolonged light-on period from that obtained at the end of that period.

**Fig. 4:**
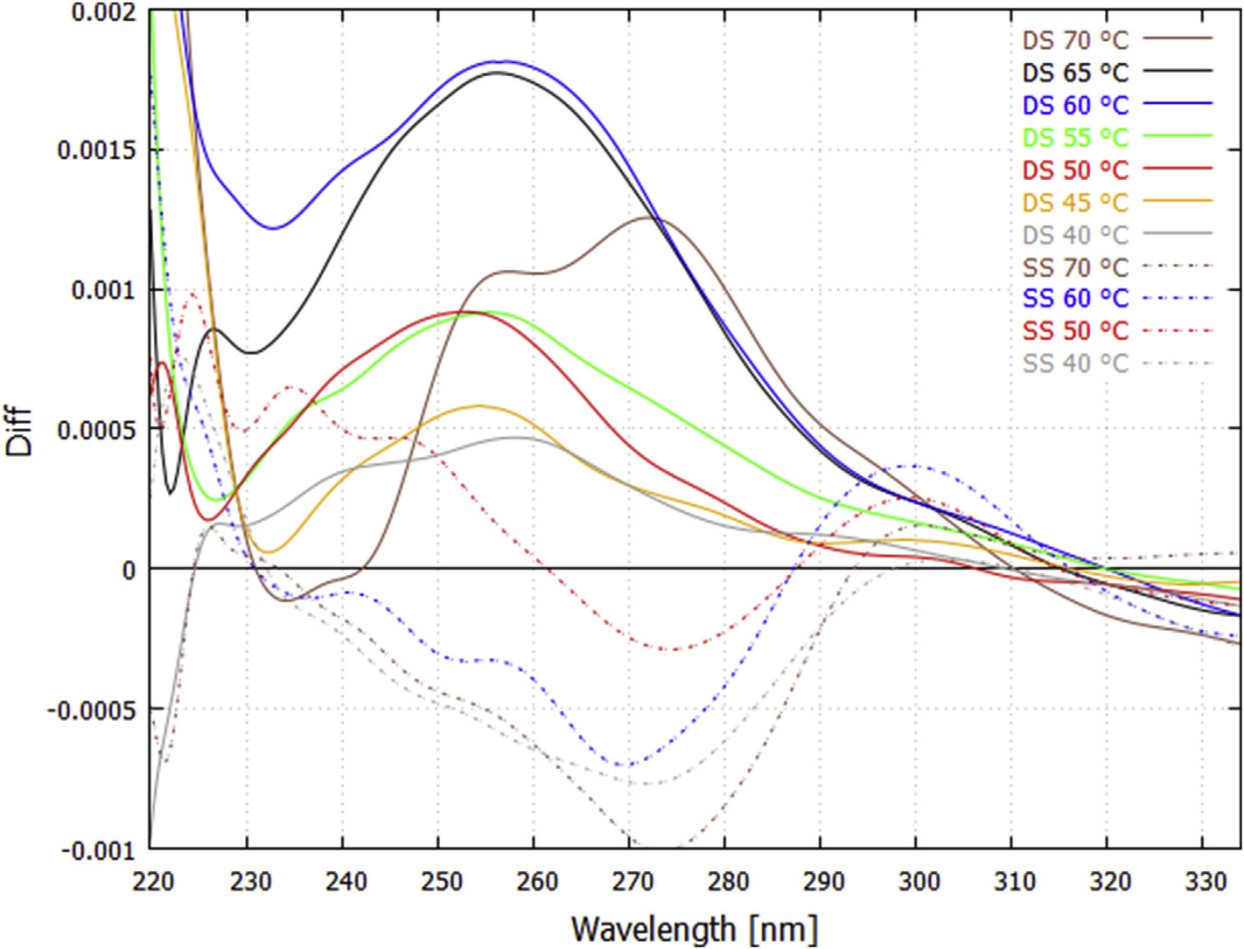
Difference spectra obtained for UVC light-induced denaturing at 70, 65, 60, 55, 50, 45, and 40 °C for double strand (DS – upper solid curves) 25 bp DNA, and at 70, 60, 50, and 40 °C for single strand (SS - lower dashed curves). The average of the two light-on runs at a given temperature (see Fig. 3) is plotted and the data have been smoothed with a 2000 point Bezier function. An increase in extinction over the light-on period is observed for the double strand DNA (attributed to light-induced denaturing), while a decrease at ~273 nm is observed for single strand DNA (attributed to dimer formation). For the double strand DNA at 70 °C, light-induced denaturing appears to be operating predominantly over G-C bonds, as suggested by the two peaks in the curve at 254 nm and 272 nm, which correspond to the maximum absorption of guanine and cytosine respectively.

In Fig. 4 we plot the *UVC light-induced* denaturing difference spectra for the double strand DNA at particular fixed temperatures (70, 65, 60, 55, 50, 45 and 40 °C) and compare these with those obtained for the single strand DNA at (70, 60, 50 and 40 °C). Exposure of double strand DNA to UVC light for a one hour period increases the absorption over the 240–290 nm region (upper solid curves of Fig. 4) and gives similar (but not identical) wavelength dependence as the difference spectra for thermal-induced denaturing at the same temperature (Fig. 1). For single strand DNA there appears to be an inverse effect, i.e. a decrease rather than an increase in the extinction over this wavelength region (lower dashed curves of Fig. 4). We attribute this to the formation of dimers (e.g. TC, CC, AA or TA* -- TT dimers were excluded by design of the oligo -- see Discussion and Supplementary Content) since the maximum decrease in absorption occurs at ∼ 270 nm, which is what Setlow and Carrier have identified as dimer formation [28].

**Fig. 5:**
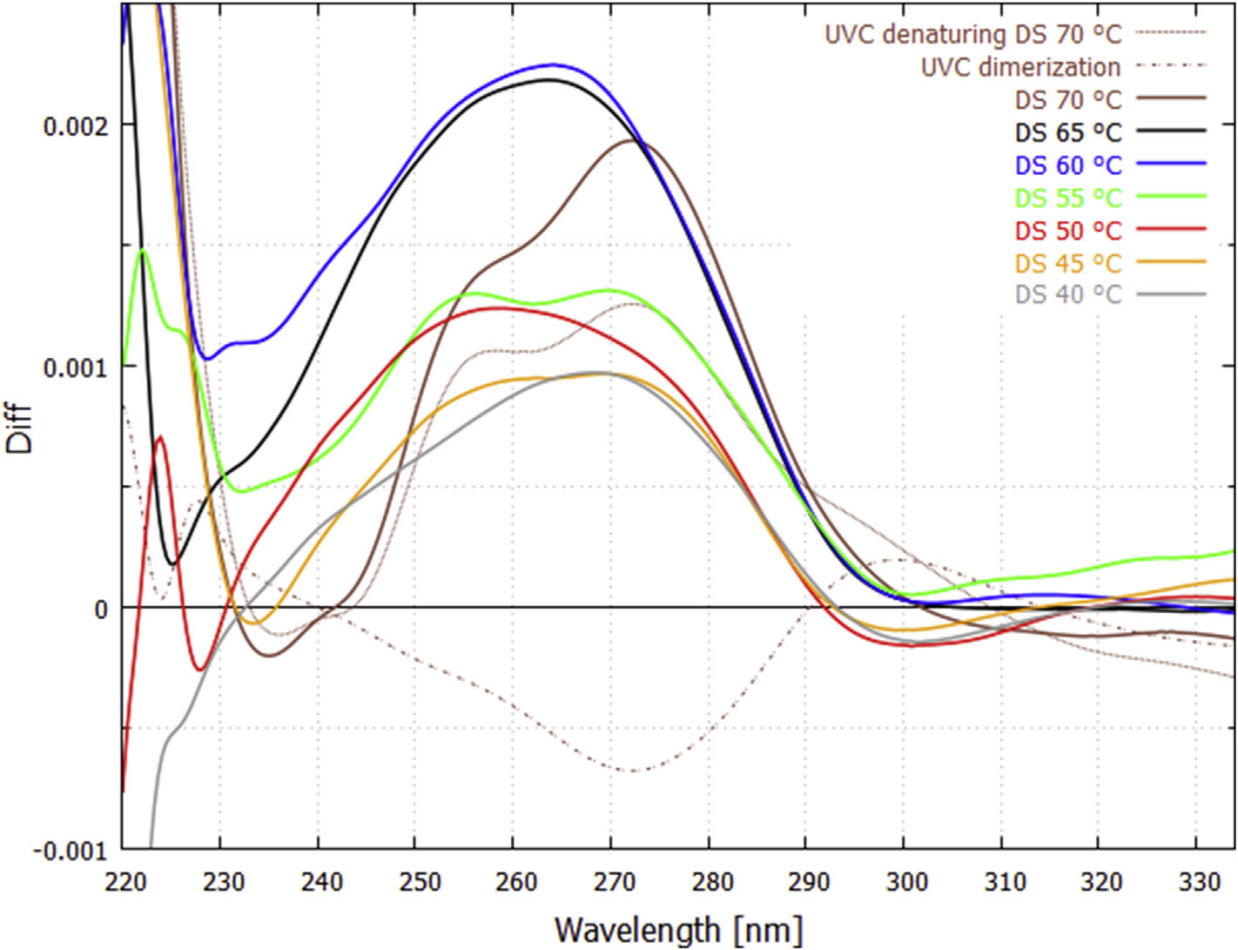
UVC light-induced denaturing curves corrected for dimer formation. An example is given for 70 °C (plum colored lines), the inverse of the lower dotted curve (reduction in extinction due to dimer formation obtained by averaging over the four dashed lines in Fig. 4) is added to the upper solid curve (increase in extinction due to UVC denaturing) to give the corrected denaturing thick plum colored line. Only the final result is given for the other temperatures but the constituent curves appear in Fig. 4.

Dimer formation during exposition to UVC light is also occurring for the double strand DNA, reducing the extinction most strongly at 273 nm, but this is overshadowed by the hyperchroism due to UVC-induced denaturing. In a first approximation, we can correct for dimer formation by adding the inverse of the dimer formation curves for the single strand DNA to the denaturing extinction curves for the double strand DNA (i.e. adding the inverse of the dashed lines to the solid lines in Fig. 4 at the corresponding temperature) giving the results shown in Fig. 5.

**Fig. 6:**
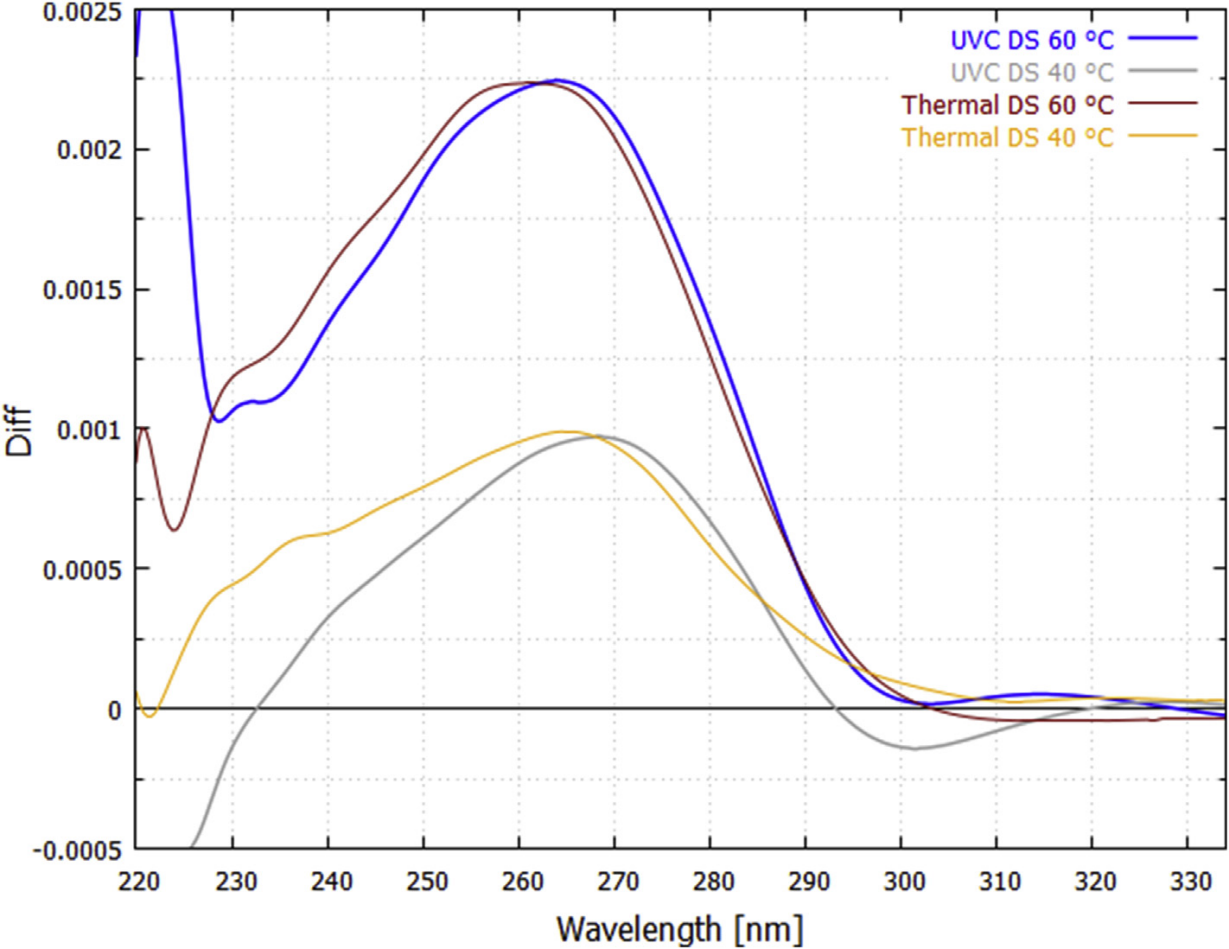
Comparison of the wavelength dependence of UVC photon-induced denaturing (Fig. 5) with thermal-induced denaturing (Fig. 1) for the 25 bp synthetic DNA at 60 and 40 °C. The thermal-induced difference curves correspond to a 3 °C bin centered on the particular temperature and they have been normalized by multiplying by factors 0.265 and 0.335 for 60 and 40 °C respectively. The data have been smoothed with a 2000 point Bezier function.

These difference curves for UVC light-induced denaturing corrected for dimer formation can be compared to the difference curves for thermal-induced denaturing (Fig. 1) at a particular temperature. The result, for temperatures 60 and 40 °C, is given in Fig. 6. Not all the light-induced difference curves are identical to the thermal-induced difference curves and this can be attributed to different denaturing mechanism operating in the two cases. For example, at 70 °C the light-induced difference curves show the prominent peaks of guanine and cytosine absorption (Fig. 5), suggesting that, at this temperature, UVC light-induced denaturing is acting predominantly over the G-C base pairs, while this is not true for thermal-induced denaturing (Fig. 1).

**Fig. 7:**
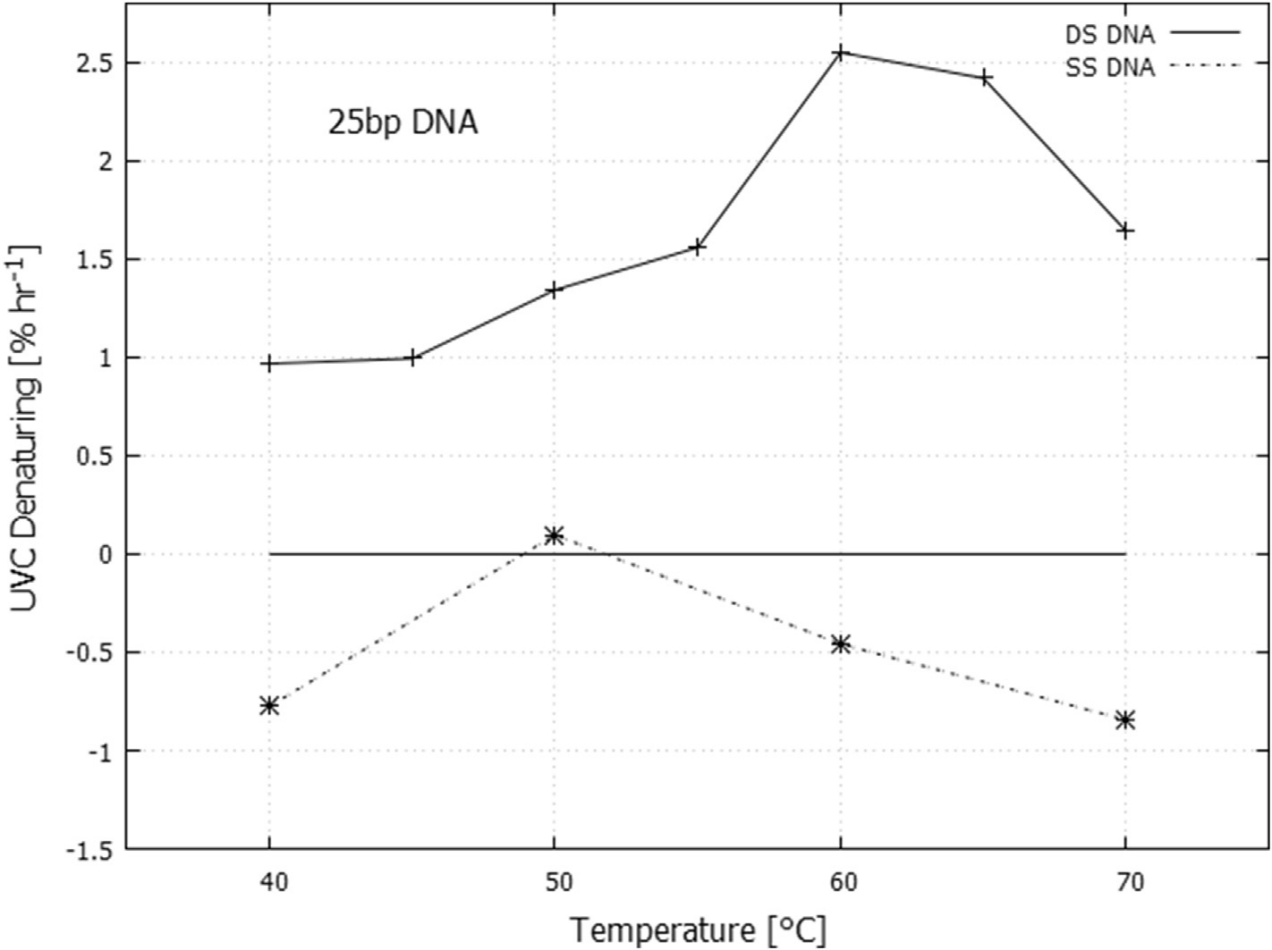
Rate of UVC light-induced denaturing as a function of temperature for double strand 25 bp DNA (+) and rate of reduction in absorption due to dimer formation for single strand 25 bp DNA (*). Values plotted are integrals from 235 to 295 nm of the difference curves given in Fig. 5 normalized to that obtained for complete thermal denaturing.

The temperature dependence of the rate of UVC light-induced denaturing can be determined by integrating the difference curves (Fig. 5) over the wavelength region from 235 to 295 nm and then scaling the result to that obtained for the total temperature-induced denaturing (assuming a linear relation between hyperchromicity and denaturing). The result is given in Fig. 7.

**Fig. 8:**
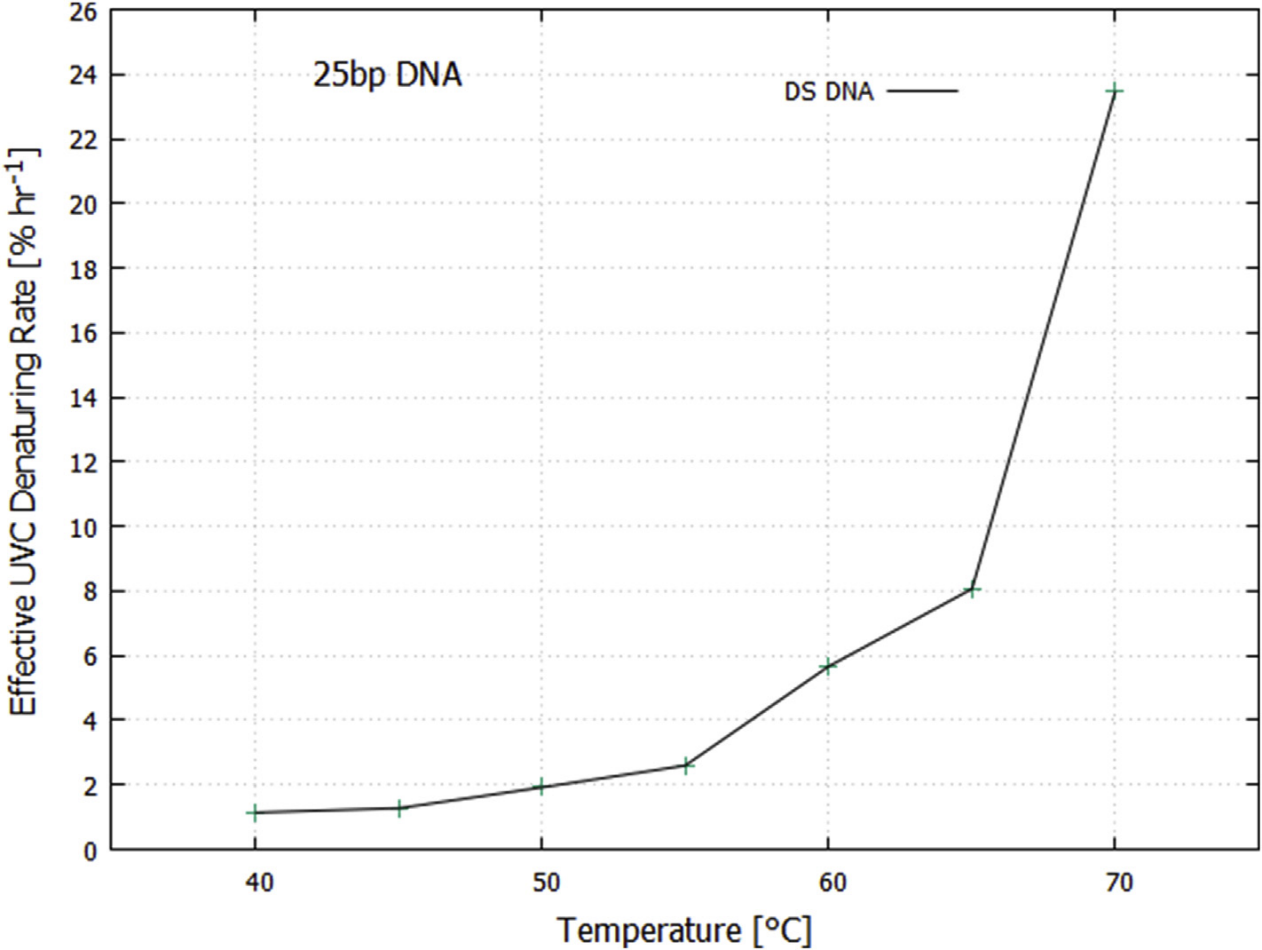
Effective rate of UVC light-induced denaturing of still bonded base pairs for the double strand 25 bp DNA as a function of temperature.

Since the number of intact intra-strand hydrogen bonds decreases with increasing temperature, the effective rate of UVC light-induced denaturing of the remaining bonded base pairs at a given temperature can be obtained by dividing the results given in Fig. 7 by the fraction of bonded base pairs remaining at a given temperature (obtainable form Fig. 2). The result is given in Fig. 8.

### 3.2 Renaturing

**Fig. 9:**
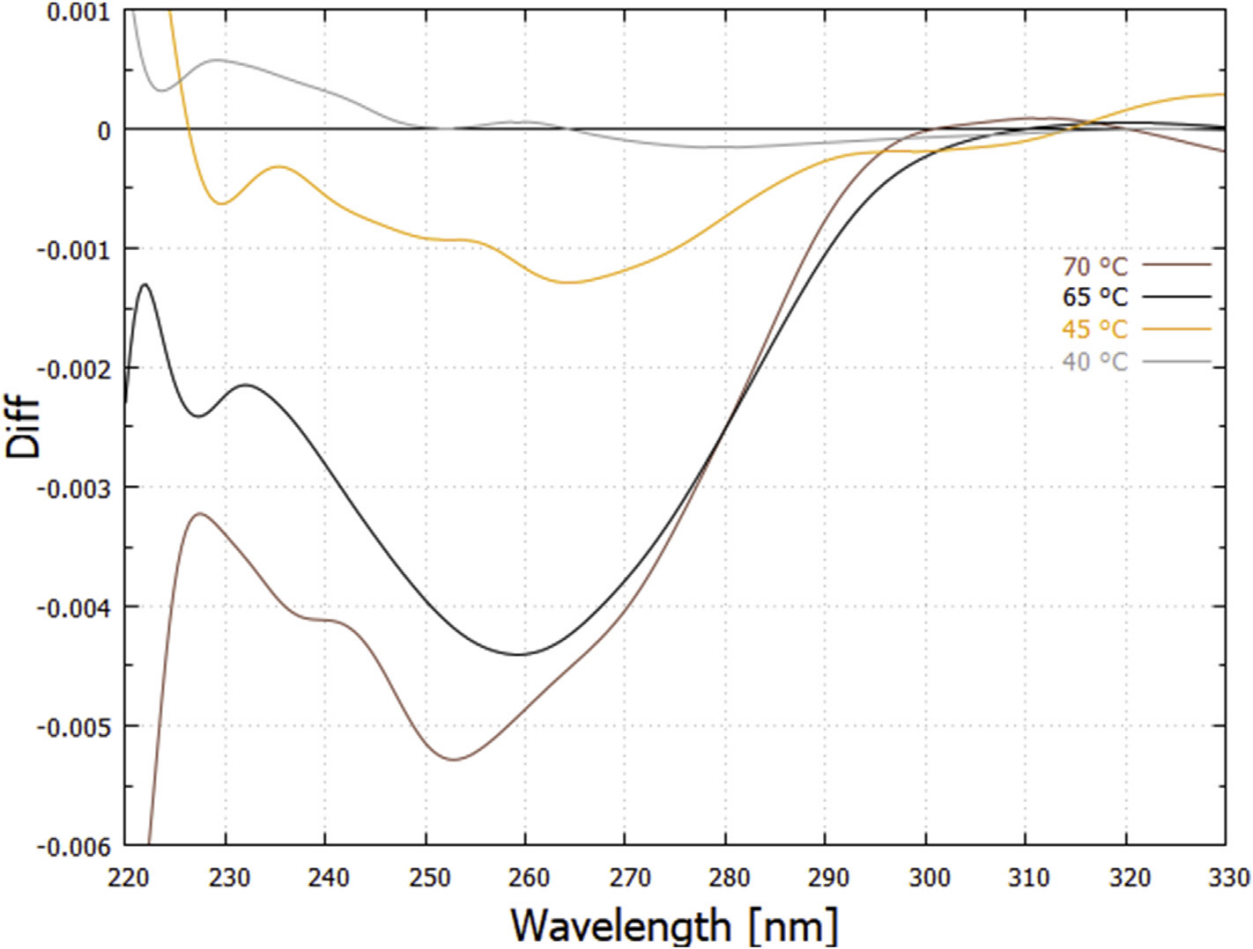
Renaturing difference curves obtained over the light-off periods for selected fixed temperatures. As expected, the wavelength dependence of the renaturing curves is similar to the inverse of that for the thermal denaturing curves (Fig. 1, lower curves). At 70 °C, renaturing appears to be operating predominantly over G-C bonds, as suggested by the two peaks in the curve at 254 nm and 272 nm (corresponding to the maximum absorption of guanine and cytosine respectively). This was also observed for UVC light-induced denaturing at 70 °C (Fig. 4). The data have been smoothed by a Bezier 2000 point function.

**Fig. 10:**
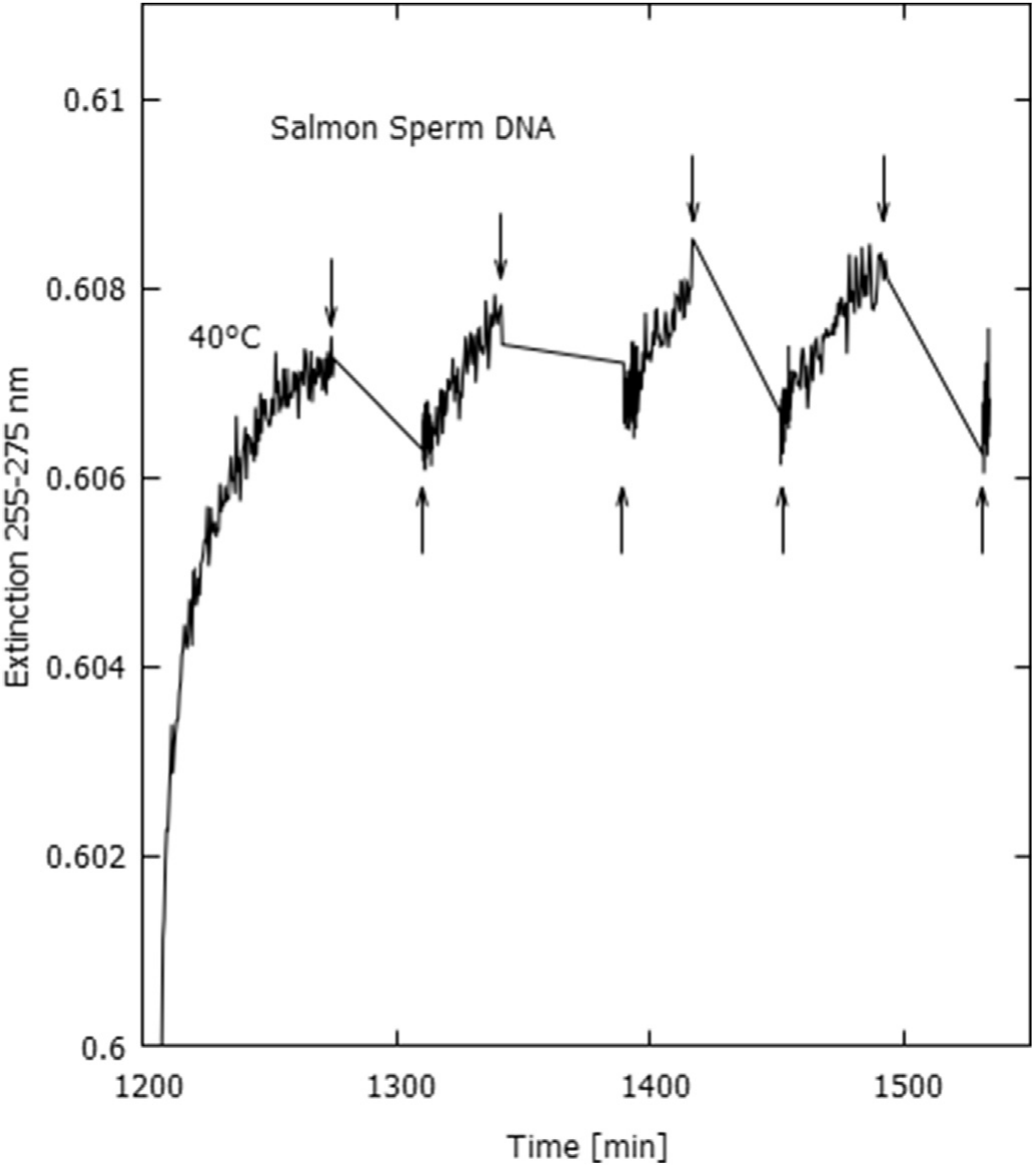
An increase in extinction (averaged over 255–275 nm) is observed and is attributed to UVC light-induced denaturing beginning at the ½ hour light-on periods (up arrows), and a decrease in extinction attributed to renaturing beginning at the ½ hour light-off periods (down arrows), for Salmon sperm DNA in purified water at 40 °C. The low intensity deuterium light was used to obtain these results.

Renaturing of completely separated DNA proceeds via chance encounter of a few complementary base pairs and then rapid re-zipping of the remaining pairs. It is thus second order in the concentration but there is also a volume or steric effect since, especially for longer DNA, the folding of single strands makes some base pairs less accessible to first encounter. Renaturation is thus a diffusion plus steric limited process with some peculiarities that depend on the nucleotide complexity, the ionic nature of the solvent, and also the viscosity of the solvent [33]. Renaturing is readily observeable as a decrease in extinction during the light-off periods for of 25 bp DNA (Fig. 3) and for salmon sperm DNA (Fig. 10). The difference spectra taken over the light-off periods for the 25 bp DNA, Fig. 9, show the expected wavelength dependence, just the inverse of the thermal-induced denaturing curves (Fig. 1).

Evidence of UVC light-induced denaturing was also observed for the natural salmon sperm DNA in purified water (Fig. 10). In this case, an important part of the light-induced denaturing is due to the formation of reversible TT dimers (see Discussion section and Supplementary Content).

For the salmon sperm DNA in purified water at 40 °C (at the start of its denaturing curve), a 1/2 hour light-on period is sufficient to increase the extinction averaged over the 255–275 nm region by 0.002 units (Fig. 10), which corresponds to 1.7% of the total thermal-induced hyperchromicity at 90 °C, implying a UVC light-induced denaturing rate of approximately 3.4% per hour.

As a further experimental control, we carried out an identical experiment by replacing the UVC deuterium lamp with the control halogen-tungsten lamp with output wavelengths only in the visible (>450 nm). In this case, for 48 bp DNA, no increase in the extinction (which would only be observable as a scattering component) was found during the light-on periods and no decrease was found during the light-off periods (Supplementary Content, Fig. S3). This confirms that the denaturing effect found with the deuterium lamp can be attributed to its UV component.

## 4 Discussion

### 4.1 Comparison of our experiment conditions with Archean conditions

It is pertinent to compare our experimental conditions of light, temperature, and DNA concentrations with those likely during the prebiotic period of the early Archean (∼3.85 Ga). Sagan [14] has calculated an integrated UVC flux during the Archean over the 240–270 nm region where DNA absorbs strongly (see Fig. 1) of 3.3 W/m^2^. Our light on sample was estimated to have a flux over this same wavelength region of 2.9 W/m^2^ for the low intensity lamp and 66.9 W/m^2^ for the high intensity lamp. However, the beam volume on sample was only 0.107 cm^3^ while the total volume of the sample was 2.0 cm^3^ (2 ml) for the salmon sperm sample and 3.5 cm^3^ (3.5 ml) for the synthetic 25 bp sample, and uniform mixing of the DNA sample due to the magnetic stirrer ensured homogeneous passage of the DNA through the whole volume, giving an effective UVC light flux of only 0.155 W/m^2^ for the salmon sperm sample and 2.045 W/m^2^ for synthetic 25 bp sample, approximately 1/20 and 2/3 respectively of what it may have been at the ocean surface during the Archean.

Oxygen isotope geological data suggest that at 3.85 Ga the Earth was kept warm by a CO_2_ and CH_4_ atmosphere, maintaining average surface temperatures around 80 °C [18, 19, 34] and falling to 70 ± 15 °C by 3.5–3.2 Ga [35]. Of course, Polar Regions would have been colder and Equatorial Regions warmer. Our results, obtained at temperatures 40, 45, 50, 55, 65, and 70 °C, indicate that UVC light-induced denaturing increases with temperature (Figs. 7 and 8).

Miller [36] has estimated adenine concentrations of 15 μM in the prebiotic soup using calculations of photochemical production rates of prebiotic organic molecules determined by Stribling and Miller [37]. Although these estimations have been considered as overly optimistic by some, we note that Miller did not consider non-equilibrium thermodynamic routes to nucleotide proliferation through photon dissipation [5, 9]; for example, the Ferris and Orgel route to pyrimidine production through UVC light on HCN [9, 38], or the purine production in UVC irradiated formamide [39], nor did he consider the existence of an organically enriched sea surface skin layer [40, 41]. Therefore, Miller's determinations of nucleotide concentrations at the beginnings of life may, in fact, be overly conservative rather than overly optimistic. Nucleobase concentrations in our sample DNA were of the order of 50 μM.

The above comparisons, particularly our lower on-sample UVC net light flux compared to estimates of the surface flux during the Archean, and the fact that our DNA samples were not continuously exposed to the UVC light because of the stirrer cycling the DNA through the narrow light beam, suggest that our determined UVC induced denaturing rates should probably be considered as a conservative lower limit to rates that could have occurred on the Archean ocean surface at the origin of life.

Finally, hydrogen bonding between bases and base stacking interaction is maximal at neutral pH (our conditions). Lower DNA melting temperatures are found only for either very acidic or very alkaline conditions [42]. However, UVC light-induced denaturing may be more sensitive to pH than temperature-induced denaturing if electron transfer or deprotonation is involved (see following subsection). The ocean surface was probably more acidic during the Archean (pH 6–6.5) due to larger atmospheric CO_2_ concentrations [43]. Experiments are therefore being prepared to test the pH dependence of UVC light-induced denaturing.

### 4.2 Mechanisms of UVC-induced denaturing of DNA

We have not yet identified the main mechanism/s responsible for the observed UVC light-induced denaturing, however, the following processes are possible candidates in order of plausibility;

1. The UV-induced formation of cyclobutane pyrimidine dimers (CPDs) and their (6-4) adduct products is known to lead to local denaturing. Our synthetic DNA were designed not to have adjacent thymines, however, other pyrimidine, purine, or purine/pyrimidine (e.g. TC, CC, AA or TA*) dimerizations may occur [44, 45, 46]. The sum of the yields of all photoproducts (including the TTs) is less than 10^−2^ mol einstein^−1^ [44,46]. Splitting of the dimers (photo-reversion) for the most important of these (TTs and TCs) peak at 239 nm and the cross sections are significantly larger than those for dimer formation over a large portion of our wavelength region of interest [28, 47] (see Supplementary Content). Although we have not found data in the literature regarding reversion of AA or TA* dimers, Agasty et al. [45], have determined that on route to the formation of TA*, the bases remain for some time as cyclobutane adducts. It is plausible that during this time these could also be liable to light-induced reversion. A calculation of the convolution of our incident light spectrum with the measured wavelength dependent spectra for dimerization and monomerization (photo-reversion) of Garcès and Davila [47] indicates that the stationary state ratios of dimerized TTs to all T T adjacents would be approximately 14.5%, and same calculation for TC dimers gives 2.6%. Since our synthetic 25 bp DNA did not have any adjacent TTs, and assuming similar cross sections for dimerization and monomerization for the CC, AA and TA* dimers as for TC, and assuming a linear dependence on time to reach the stationary state of 2.6% dimers, leads to a determination of denaturing due to dimer formation of 0.054 % hr^−1^ for our experimental conditions on 25 bp DNA (see Supplementary Content), at least an order of magnitude smaller than our measured UVC light-induced denaturing rate of 2.5 % hr^−1^ at 60 °C (Fig. 7). For the natural salmon sperm DNA which does contain TT adjacent bases, we calculate a denaturing rate due to dimer formation of about 0.61 % hr^−1^, smaller than our measured rate of 3.4% hr^−1^ at 40 °C. We therefore conclude that some other photon-induced mechanism must also be operating.
The much larger cross section for monomerization (dimer splitting) with respect to dimerization over most of the wavelength region of interest (220–290 nm), and the fact that the dimerization cross section decreases after denaturing at high temperature [48, 49], leads us to conclude that any denaturing due to the formation of known photoproducts (including the TTs), should be considered as a viable, although not the dominant, mechanism for light-induced denaturing since the “damage” is reversible and the great majority of short (∼25 bp) strand DNA will be free of such photoproducts in the denatured state.
2. In UV-induced electron-driven proton transfer (EDPT), the absorption of a 260 nm photon on a DNA base leads to a charge transfer excited state that decays rapidly (sub picosecond) and without radiation to the ground state by transferring a proton from one base to another [50]; either to its hydrogen-bonded base pair, or to an adjacent neighboring base on the same strand [51]. If the transfer is to an adjacent base which happens to be in a dimer formation, then this process can lead to its monomerization [52]. If the transfer is to its hydrogen-bonded partner, it becomes a diradical pair (for example, a proton shifting from oxygen to nitrogen in the double-well hydrogen bonding potential between oxygen and nitrogen) affecting the strength of the hydrogen bond. Since the local electrostatic environment is changed by the transfer of a proton, this leads to other collective effects such as changing the delocalized electron distribution on the bases, resulting in a weakening of the base stacking interaction. In other hydrogen-bonded systems, it has been shown that a single proton transfer can initiate other proton transfer reactions in a sort of chain reaction [53, 54]. This may provide a collective mechanism for UVC-induced denaturing in DNA at high temperature.
3. The one photon quantum yield for ionization at 266 nm for double-strand (dA)20•(dT)20 in water is (1.1 ± 0.3)×10^−3^ [55]. This process may contribute to our measured UVC induced denaturing since delocalized pi-bonding electrons may be removed, thereby debilitating the stacking interaction. Furthermore, deprotonation of the DNA radical in water may subsequently occur after ionization. The DNA radicals in water decay with a half-life of 4 ms at pH 7 and room temperature [55], sufficient time to contribute to denaturing of short strand DNA [56].
4. In the ultra-rapid dissipation of the electronic excitation energy of a nucleobase into local heat, a conical intersection must be reached where the vibrational states of the electronic ground state become degenerate in energy with the vibrational states of a nuclear coordinate deformed electronic excited state. Reaching this deformed configuration requires the puckering of the C_2_ atom (Adenine) or the C_6_ atom (Guannine), and the out-of-plane distortion of the NH_2_ groups of the excited base (adenine, guanine, and cytosine) [57, 58, 59]. These could lead to base pair hydrogen bond breaking and associated charge redistribution, resulting in a weakening of the base stacking interaction.
5. Finally, another phenomenon which could provide a route to UVC-induced denaturing is the observation of long-lived and delocalized (on more than two bases) excited states in DNA. Such energy delocalization may have played an increasingly important role as DNA grew in size or in high salt environments since these long-lived excited states are more common in long DNA and strong ionic environments [60].

These phenomena, combined with a possible local entrapment of the heat of photon dissipation (for example, for DNA confined within lipid vesicles [13]), may have provided a mechanism during the Archean for the successive breaking of hydrogen bonds with each photon absorption event, particularly at frayed ends of the double helix, eventually denaturing the double helix into single strands.

### 4.3 Other experimental evidence

The only other evidence that we are aware of for UV-induced denaturing are the studies of Hagen et al. [61] on the UV inactivation of double helix DNA priming ability in the RNA polymerase system for RNA synthesis (denatured DNA cannot prime this system) using calf-thymus DNA under high doses of irradiation at 253.7 nm (100 ergs s^−1^ mm^−2^, approximately 64 times our UVC integrated on-sample irradiation of 1.55 ergs s^−1^ mm^−2^ for the low intensity lamp). They found an initial increase in absorption for their native double strand DNA after being irradiated for one hour, probably due to hyperchromicity resulting from denaturing more than offsetting any decrease in absorption due to CPD formation or other photo-damage. The first hour of irradiation also led to a marked decrease in the relative hyperchromic effect of the temperature-induced melting transition curves taken after the irradiation, which the authors concluded was due to denaturing occurring during initial UV irradiation [61] (renaturation during exposition to irradiation was prevented by the addition of formaldehyde [62]). Furthermore, during the first two hours of irradiation only a small shift of the melting temperature was found, indicating that only minor photo-damage occurred. After two hours of irradiation, their double helix sample appeared to be completely denatured since the UVC absorption versus accumulated irradiated time curve followed exactly that of single strand irradiated DNA [61]. After 10 hours of irradiation, the samples were almost completely destroyed by photo-damage as evidenced by a much reduced absorption within the 220–300 nm region (their experiments did not allow for photo-reversion of dimers to monomers since shorter wavelengths which induce reversion were not available from their low pressure mercury lamp). This behavior was found to be in contrast to X-ray irradiation which caused no discernible denaturing but did cause extensive photo-damage with consequent loss in UVC absorption [61].

### 4.4 Enzyme-less extension of DNA

Regarding the second part of the UVTAR mechanism, that of enzyme-less extension during dark and cooler, overnight periods, or during cooler early morning periods, there exists experimental evidence, employing chemical activation of the nucleotides, indicating that long random sequence linear DNA oligonucleotides can be template synthesized from a random pool of short oligonucleotides with high fidelity (with fidelity increasing with temperature) without polymerase [63]. In our proposed UVTAR scenario, UVC light activation of the nucleotides would replace chemical activation.

### 4.5 Acquisition of homochirality

The UVTAR mechanism [2, 3, 6] described above suggests a dissipation-replication relation since those DNA sequences and their complexes which captured and dissipated photons into heat most efficiently would most readily denature and thus be more available for template replication. This provides a mechanism for differential proliferation at the very beginnings of life with a clear non-equilibrium thermodynamic foundation; that of increasing dissipation.

Since DNA (and RNA) display a negative (for right-handed oligos) circular dichroism at their maximum absorption at 260 nm, at the center of the Archean atmospheric window [11], and given the empirically established excess of a particular circular polarization (right- or left-handed, depending on the hemisphere) of submarine light at the ocean surface in the late afternoon [64, 65] when surface water temperatures were highest and thus most conducive to photon-induced denaturing (Fig. 8), the symmetry between the amount of morning and afternoon photon-induced denaturing would be broken, giving greater replication probability to DNA oligos of a particular handedness depending on hemisphere, thereby providing a plausible explanation for the emergence of homochirality in life [11].

Since RNA has very similar optical and chemical properties as DNA, it is plausible that such UVTAR, homochirality, and information acquisition, mechanisms driven by photon dissipation would have operated similarly, and probably simultaneously, over RNA.

## 5 Conclusions

Our experimental results;

1) the increases in extinction during the light-on periods and decrease during the light-off periods observed for double strand DNA but not for single strand DNA (Figs. 3 and 10),
2) the shape of the difference spectra for double strand DNA corrected for dimer formation obtained over the light-on periods which has a wavelength dependence very similar to thermal-induced denaturing (Fig. 6),
3) the shape of the UVC photon-induced difference spectrum for the single strand DNA (Figs. 4 and 5) showing no increase in absorption over the UVC light-on periods, but, in fact, showing a decrease with a maximum at that identified as indicating dimer formation (Fig. 4),
4) the shape of the light-off renaturing curves for double strand DNA (Fig. 9) having the form of the inverse of the temperature induced denaturing curves,
5) the null result obtained using the visible light control (Fig. S3),

all provide strong evidence that UVC light-in the range of 220–290 nm can induce DNA denaturing and that renaturing at the same fixed temperature occurs during the dark periods.

Under our broadband wavelength light conditions, exposure to UV light causes little permanent damage to DNA since cyclobutane pyrimidine dimers are reversible at available shorter wavelengths [47] and strand breaks are very rare (see Supplementary Content). Corroborating this is the fact that we clearly observe renaturing in the light-off periods (Fig. 9), and studies by others with UVC light at much higher intensities on DNA show little affect on the melting temperature [61]. Nucleobase disassociation energies [66] are higher than those of the photons available in our experiment or at Earth's surface during the Archean.

The magnitude of the observed UVC photon-induced denaturing is sufficient to lend plausibility to proposals for the accumulation of homochirality and information content in DNA through photon dissipation [2, 3, 11, 12].

Complexation of DNA and RNA with other “antenna” molecules would have facilitated photon dissipation and replication despite the cooling of the ocean surface and the eventual attenuation of the UVC light due to the accumulation of oxygen from oxygenic photosynthesis, leaving UVC dissipation to be delegated to life derived ozone in the upper atmosphere.

Considered from within the frameworks of the thermodynamic dissipation theory of the origin and evolution of life [1, 2, 3, 4, 5, 6, 7, 8, 9, 10, 11, 12, 13], our results suggest a non-equilibrium thermodynamic imperative for an origin of life. All biotic and coupled biotic-abiotic evolution would have been driven by increases in the entropy production of the biosphere [6, 7, 8], principally by increasing the global solar photon dissipation rate. Life would have started out dissipating in the long wavelength UVC region before gradually evolving dissipation towards the visible where photon fluxes were greater but biosynthetic pathways were necessarily more complex because of insufficient free energy in individual photons for directly reconfiguring carbon covalent bonds. By associating, in this manner, molecular synthesis, replication, and complexation over evolutionary time with increasing photon dissipation, "replication first" and "metabolism first" hypotheses can be reconciled. Photon dissipation may have been the most primitive and, as today, the most extensive, of all metabolisms performed by life.

## Declarations

### Author contribution statement

Karo Michaelian: Conceived and designed the experiments; Performed the experiments; Analyzed and interpreted the data; Contributed reagents, materials, analysis tools or data; Wrote the paper.

Norberto Santillán Padilla: Conceived and designed the experiments; Performed the experiments; Analyzed and interpreted the data; Contributed reagents, materials, analysis tools or data.

### Funding statement

This work was supported by Direccion General de Asuntos del Personal Académico, DGAPA IN-103113 and IN-102316, and CONACyT via financial support to Norberto Santillán Padilla.

### Competing interest statement

The authors declare no conflict of interest.

### Additional information

No additional information is available for this paper.

## Appendix A Supplementary data

Supplementary Content Supplementary Content
