# Supplementary material for: UVC photon-induced denaturing of DNA: A possible dissipative route to Archean enzyme-less replication
Source: Heliyon. 2019 Jun 18;5(6):e01902. doi: 10.1016/j.heliyon.2019.e01902 (PMC6584779; doi:10.1016/j.heliyon.2019.e01902)
Supplement: Supplementary Content [file mmc1.docx]

Supplementary Content for: **UVC Photon-induced denaturing of DNA: A possible dissipative route to Archean enzyme-less replication**

Karo Michaelian^1*^ and Norberto Santillán Padilla^2^

^1^ Department of Nuclear Physics and Application of Radiations, Institute of Physics, UNAM. Cto. Interior de la Investigación Científica, Ciudad Universitaria, Cuidad de México, C.P. 04510.

^2^Faculty of Science, UNAM. Cto. Interior de la Investigación Científica, Ciudad Universitaria, Cuidad de México, C.P. 04510.


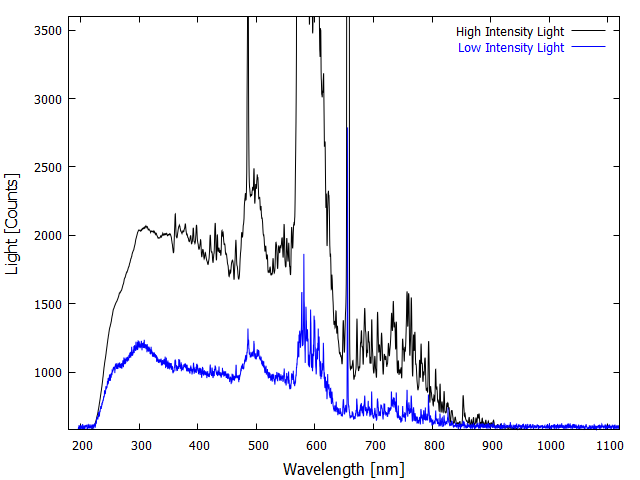


Figure S1 Light output spectra of our high and low intensity deuterium lamps as seen through the reference sample (quartz cuvette and PBS buffer but no DNA). Integration times for these graphs were 20 ms for the high intensity lamp and 100 ms for the low intensity lamp. The output of the visible halogen lamp (not shown) only starts to become significant at wavelengths greater than 450 nm.


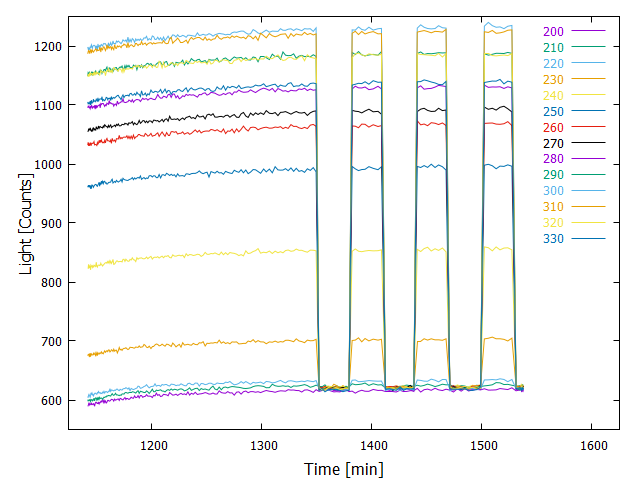
Fig. S2 The convoluted light and detector drift as a function of time (actual time in minutes since midnight) using our low intensity lamp observed through the quartz cuvette plus PBS buffer (no DNA) at 20 °C. Wavelength bins were 10 nm wide centered on the specified wavelength. The shortest wavelength traces begin at the bottom and increase upwards. Little change in the variation over wavelength is seen. Drift is not affected by the change in the shutter condition; the first of 3 light-off conditions begins at about 1350 min.


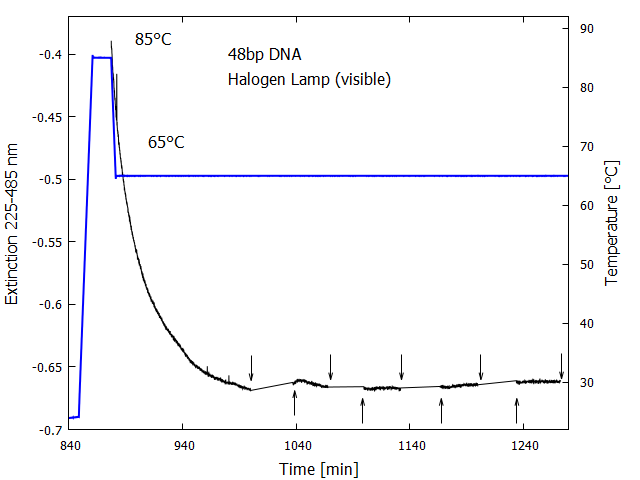


Fig. S3 Extinction for 48 bp synthetic double strand DNA under our halogen visible lamp (> 450 nm). The up and down arrows mark the beginning of the light-on and -off periods respectively. The decrease in extinction (only observable in scattering) correlates with a decrease in temperature (blue line, right scale) due to renaturing as the temperature is lowered. However, at a fixed temperature of 65 °C, no increase in extinction is observed during the light-on periods, and no decrease in extinction is observed during the light-off periods, ostensibly because visible light cannot induce DNA denaturing. This supports the conclusion that the increase in extinction observed during the light-on periods with the deuterium lamp (article, Figs. 3 and 10) is a UVC light-induced effect and not an instrumental artifact.

**Is cyclobutane pyrimidine dimer formation sufficient to account for our observed UVC light-induced denaturing?**

In the following we determine approximately the contribution of the formation and reversion of UVC-induced photoproducts, in particular cyclobutane pyrimidine dimers (CPDs), to our experimentally observed light-induced denaturing. We conclude, especially for the 25 bp synthetic DNA which was designed not to have adjacent thymines, that there must also exist some other light-induced mechanism contributing to the denaturing. We also show that most photo-damage to DNA is photo-reversible as long as shorter wavelengths (~239 nm) are available, which was the case in our experiments and would have been the case on the surface of Earth during the Archean.

Below we plot the dimerization and monomerization (photo-reversion) cross sections as a function of wavelength as determined by Setlow and Setlow (1962) and by Garcès and Dávila (1981) for TT dimers (figures S4 and S5 respectively), and by Garcès and Dávila for CT dimers (figure S6). For both these CPDs, the cross section for monomerization is significantly larger than that for dimerization at most wavelengths in our region of interest (220-290 nm). On the same plots, we give our deuterium lamp output in photon counts (blue line, right y-scale) as a function of wavelength, as measured for our low intensity lamp (see figure S1).


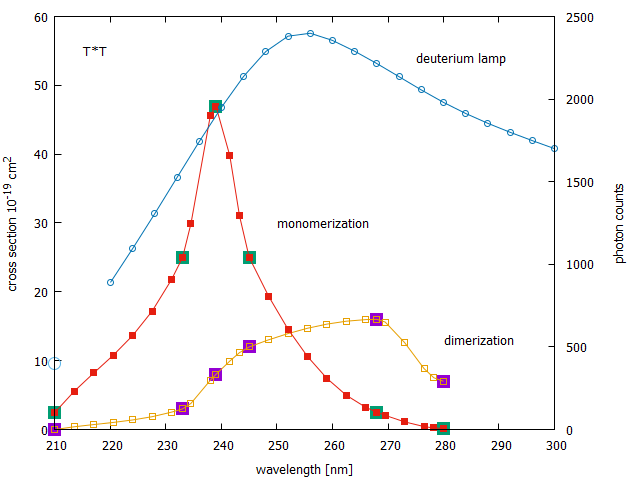
Fig. S4 The cross sections for the dimerization of adjacent thymines (formation of TT CPDs) and for the monomerization of these (reversion to monomers) as a function of wavelength. The large solid square points are the data of Setlow and Setlow (1962), while the smaller points are interpolated values obtained using a spline fitting program. Since Setlow and Setlow (1962) measured the cross sections only at wavelengths down to 239 nm, the data points at 210 and 232 nm are fictitious and included so as to make the monomerization cross section symmetric about its maximum at 239 nm and to make the dimerization cross section go to zero according to the trend of the two shortest wavelength points measured (at 239 and 245 nm). Also drawn on the graph is our deuterium lamp spectrum in this wavelength region (blue line, right axis, see figure S1).


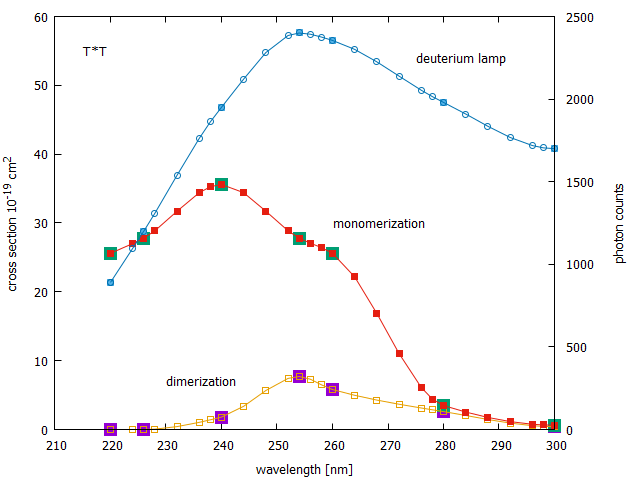


Fig. S5 Dimerization and monomerization cross sections for the CPD TT obtained by Garcès and Dávila (1981). The large solid square points are data taken from the reference, while the smaller points are interpolations obtained using a spline fitting. Since data points for wavelengths shorter than 240 nm were not measured by Garcès and Dávila, the data points at 220 and 226 nm are fictitious and included so as to maintain the trend towards zero of the data at 240 nm for the dimerization cross section, and symmetry about the peak at 239 nm was maintained for monomerization cross section. The light output of our deuterium lamp is also shown on the graph (blue line, right axis). A similar relation was found between the wavelength dependent cross sections for dimerization and monomerization of adjacent U pairs (UU) in RNA (Swenson and Setlow, 1963).


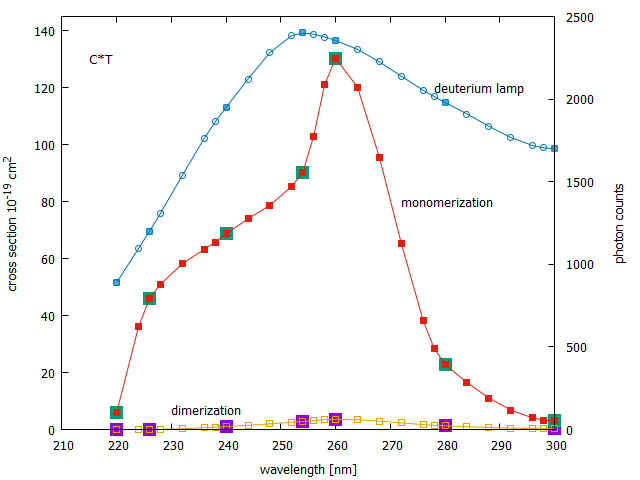


Fig. S6 Dimerization and monomerization cross sections for the CPD CT obtained by Garcès and Dávila (1981, from their table 2). The large solid square points are data taken from the reference, while the smaller points are interpolations obtained using a spline fitting. Since data points for wavelengths shorter than 240 nm were not measured by Garcès and Dávila, the points at 220 and 226 nm are fictitious and included such that the trend towards zero of the data at 240 and 254 nm was maintained. The light output of our deuterium lamp is also shown on the graph (blue curve, right axis).

The dynamics of dimer formation can be obtained from a convolution of the measured wavelength dependent cross sections for dimerization and monomerization with the incident light spectrum. For the calculation, we use the more recent data of Garcès and Dávila (1981) (figure S5) since they state that they obtained better statistics than previous determinations for the cross sections. The convolution of the dimerization cross section spectrum with that of the incident light spectrum, employing the sum of the equally spaced interpolated data points, is

$$CD=\sum_{=220}^{290} \sigma_{D}\left( \right)L\left( \right)=168252.$$

in units of 10^-19^ cm^2^, and where σ _D_ $\left( \right)$ is the cross section for TT dimer formation and L(λ) is the light output of the lamp (blue line, figure S5) obtained at the interpolated points between 220 to 290 nm.

And similarly, for monomer formation (reversion of dimers) the convolution is

$$CM=\sum_{=220}^{290} \sigma_{M}\left( \right)L\left( \right)=990807.$$

where $\sigma_{M}()$ is the cross section for monomerization (Fig. S5 red curve, interpolated points).

At the stationary state (i.e. at high incident photon flux or after a long time with a low intensity lamp), the probability of finding a given T T pair being in a dimer TT formation is therefore,

P^TT^= $CD/(CD+CM)= 168252. /(168252. + 990807.) = 0.1451$

Similarly, doing the same integrations but now for the CT dimer and monomerization cross sections given in figure S6, the probability that a given C T pair would be in a dimer CT formation in the stationary state is,

P^CT^ = *CD/(CD+CM)* = 0.0261

In summary, the probability that a given T T adjacent pair is in a dimer TT formation in the stationary state (after a long time, or under high light flux conditions) is about 14.5%, while that for CT in dimer formation is 2.6%. In lieu of available measured wavelength dependent cross sections for the other CPD dimers of even smaller quantum yield, in the calculations below, it is assumed, as a worse case scenario, that they would have the same probability for existence in the stationary state as the CT dimers (i.e. 2.6%).

**Calculation of average number of photons in the 220-290 nm region absorbed by our 25 bp DNA per second**

For the 25 bp double strand DNA we determined a concentration of 1.56 μM from its absorption value at 260 nm. From Eq. (1) of the manuscript, we have,

Extinction (averaged 220 -290 nm) = 0.3 = -log_10_ (Ld/Li)

(where Ld is the light flux detected and Li is the light flux incident), or a ratio of detected to incident light of

Ld/Li = 10^-0.3^ = 0.50

(i.e. on average over the 220-290 nm region, 50% of the incident light gets through the sample, 50% is therefore absorbed in the DNA).

We had 217 (9.4) μW on sample light flux from the deuterium high (low) intensity lamp (measured with lamp coupled to a 600 μm diameter UV/vis optical cable) integrated over the full spectrum of the lamp (see figure S1) so we estimate that we had, as an upper limit, 1/3 of this energy within the 220-290 nm region. Therefore, the amount of light absorbed by our 25 bp DNA was 0.50 x 1/3 x 217 μW = 36.2 μW, or 36.2x10^-6^ J/s x 1 eV/1.602x10^-19^ J = 22.6x10^13^ eV/s. A photon of 260 nm has 4.77 eV so this equals 22.6x10^13^eV/4.77 eV = 4.73x10^13^ photons absorbed per second. Our concentration of 25 bp DNA was 1.56 µM , or 1.56x10^-6^ x (6.023x10^23^)/ l = 9.39x10^17^ DNA / l. Our sample volume was 3.5ml so this gives 3.3x10^15^ DNA. Therefore, on average, each 25 bp DNA could be expected to absorb 4.73x10^13^/3.3x10^15^ = 1.43x 10^-2^ photons/s (i.e. one photon absorption every 70 s).

**How long does it take for our 25 bp DNA to arrive at the stationary state concentrations of dimers?**

The time it would take the DNA to reach a stationary state with respect to dimer formation and reversion would be of the order it would take for each TT pair to be converted into a pyrimidine dimer at least once. For the sake of calculation, we first assume a randomly constructed DNA of equal concentration of all the bases. The quantum efficiency for the sum of all CPD formations is approximately 10^-2^ mol einstein^-1^ at 254 nm for calf thymus DNA (Davies, 1994) and we assume it is the same for the randomly constructed DNA with equal concentration of each base type. However, since this quantum efficiency is not constant over our full wavelength region of interest (see figure S4 or S5 above), a more accurate value would be half of this, or 0.5x10^-2^ mol einstein^-1^ over our wavelength region of interest (220-290 nm).

Since, as we calculated above, it would take on average 70 s for each photon absorption event on a particular 25 bp DNA, we could expect that the average time for producing a TT dimer on a particular oligo would be 70/0.5x10^-2^ = 14000 s, or 3.9 hours. Since we have 25/16 = 1.56 T T pairs on average on a randomly constructed 25 bp DNA it would take 1.56x3.9 = 6.1 hours for every T T to be converted at least once into a TT dimer. Therefore, it would take about 6 hours to reach the stationary state of 14.5% of adjacent T Ts in a TT dimer configuration.

The average number of T T pairs on a randomly constructed 25 bp DNA is 1.56 and these would occupy 1.56x(2/25) = 12.5% of the DNA length. However, once arriving at the stationary state, only 14.5% of the T T pairs would be in dimer formation and it takes 6 hours to reach this maximum concentration of dimers. We now further assume that the formation of a dimer will lead to not only a denaturing of the two thymines, but also a weakening of the neighboring base pairs on both sides (2 other nucleotides are also affected – note that this is probably a worst case scenario since it has been determined that the formation of dimers, very surprisingly, does not affect the overall Gibb’s free energy – see Rumora et al., 2008). Therefore, the maximum rate of DNA denaturing due to this dimer production process (assuming a linear increase in dimers with time before reaching the stationary state) would be 2x12.5%x14.5%/6 hours, or 0.61% /hour. For comparison, our calculated UV-C light induced denaturing rate for the salmon sperm DNA was 3.4% per hour at 40 °C (see manuscript).

Our 25 bp synthetic DNA, however, was designed not to have T T adjacent bases. The other possible photoproducts are CC, CT, TC, TA* and AA dimerizations. The sum of the quantum yields of these 5 dimers on double strand DNA is less than 10^-3^ mol einstein^-1^ at 254 nm (Douki, 2013; Davies, 1994) or about 1/10 the TT quantum yield (for example, quantum yields are 1.4x10^-5^ and 2.0x10^-5^ for TA* and AA respectively - tables 1 and 2 of Davies (1994)). Assuming again a wavelength integrated (220-290 nm) quantum yield of ½ this value, and since we calculate that we get on average one photon absorption event per DNA every 70 s, the average time it would take to form such a photoproduct on a given DNA would be 70/0.5x10^-3^ = 1.4x10^5^ s or 38.9 hours. Since we have 25/16 = 1.56 of each of such pairs on such a constructed 25 bp DNA, it would take 1.56x38.9 = 60 hours for every such pair to be converted at least once into a dimer. Therefore, it would take 60 hours to reach the stationary state of 2.6% of each type of pair in a dimer configuration. We are assuming that all photoproducts (except TTs) have a similar relation between their production and reversion cross sections as do CT products. There does not appear to be published data with respect to the possible reversion of TA* or AA photoproducts, however, for example, reversion could occur during the time TA* products remain as cyclobutane adducts. (Agasty et al., 2010 determined that on route to the formation of TA* the bases remain for some time as cyclobutane adducts.)

These 5 distinct pairs would make up a maximum (assuming no overlap) of (5 x1.56) x (2/25) or 62% of the 25 bp DNA length. Assuming again 2 neighboring base pairs affected for every dimer, this would give a maximum denaturing rate of 2x62% x 2.6%/60 = 0.054 % hr^-1^. For comparison, our measured UV-C light induced denaturing rate was calculated to be 1.0 % per hour for our 25 bp DNA at 40 °C and 2.5% per hour at 60 °C (see article, figure 7) and therefore this is between one and two orders of magnitude larger than could be attributed to CPD dimer formation alone. We therefore conclude that some other photon-induced process leading to denaturing must be operating besides dimer formation and reversion.

**Could UV-induced strand breaks account for some of our observed UVC-induced denaturing?**

The quantum yield for double helix strand breaks on DNA are 1.4x10^-6^ at 254 nm (Görner, 1994 – Table 2). With 1.43 x 10^-2^ photons/s absorbed on our 25 bp DNA this would mean on average a given DNA would break every 1/(1.43 x 10^-2^ photons/s x 1.4x10^-6^) = 5.0x10^7^ s or every 13,888 hours under our intense deuterium lamp and we can therefore safely neglect this process as contributing, in any measurable way, to the UVC-induced denaturing.

**References:**

Asgatay, S. et al. UV-Induced TA Photoproducts: Formation and Hydrolysis in Double-Stranded DNA. *J. Am. Chem. Soc.* **132**, 10260-10261 (2010).

Davies, R. J. H. Ultraviolet Radiation Damage in DNA. *Biochem. Soc. Trans*. **23**, 407-418 (1994).

Douki, T. The variety of UV-induced pyrimidine dimeric photoproducts in DNA as shown by chromatographic quantification methods. *Photochem. Photobiol. Sci*., **12**, 1286-1302 (2013).

Garcès F. & Davila C. A. Alterations in DNA irradiated with ultraviolet radiation---I. The formation process of cyclobutylpyrimidine dimers: cross sections, action spectra and quantum yields. *Photochem. Photobiol.* **35**, 9-16 (1982).

Görner, H. Photochemistry of DNA and related biomolecules: quantum yields and consequences of photoionization, *Journal of Photochemistry and Photobiology B: Biology* **26**, 117-139 (1994).

Rumora, A. E. et al. Thymine dimer-induced structural changes to the DNA duplex examined with reactive probes. *Biochemistry* **47**,49, 13026-13035 (2008). doi:10.1021/bi801417u

Sander, M. U., Luther, K. & Troe, J. On the Photoionization Mechanism of Liquid Water. *Berichte der Bunsengesellschaft für physikalische Chemie*, **97**, 953–960 (1993). doi:10.1002/bbpc.19930970802

Setlow, R. B., & J. K. Setlow***.*** Evidence that ultraviolet-induced thymine dimers in DNA cause biological damage. *Proc. Natl. Acad. Sci. USA,* **48,** 1250-1257 (1962).

Swenson, P.A. & Setlow, R.B. Kinetics of dimer formation and photohydration in ultraviolet-irradiated polyuridylic acid, *Photochemistry and Photobiology* **2,** 419-434 (1963) DOI: 10.1111/j.1751-1097.1963.tb08899.x
